# Outcome saliency modulates behavioral decision switching

**DOI:** 10.1038/s41598-020-71182-9

**Published:** 2020-08-31

**Authors:** Sai Sun, Rongjun Yu, Shuo Wang

**Affiliations:** 1 Center for Studies of Psychological Application, Key Laboratory of Mental Health and Cognitive Science of Guangdong Province, School of Psychology, South China Normal University, Guangzhou, 510631 China; 2 Department of Psychology, National University of Singapore, Singapore, 117570 Singapore; 3 Department of Chemical and Biomedical Engineering and Rockefeller Neuroscience Institute, West Virginia University, Morgantown, WV 26506 USA

**Keywords:** Attention, Decision

## Abstract

Goal-directed decision making often requires evaluating the outcomes of our decisions, assessing any gains or losses, learning from performance-related feedback, and deciding whether to alter our future decisions. However, it is unclear how these processes can be influenced by the saliency of an outcome (e.g., when one aspect of the outcome is accentuated more than another). Here we investigated whether decision strategies changed when certain aspects of the task outcome (win/loss or correct/incorrect) became more salient and how our brain encoded such saliency signals. We employed a simple two-alternative forced choice gambling task and quantified the frequency at which participants switched decisions to an alternative option in the subsequent trial after receiving feedback on their current selection. We conducted three experiments. In Experiment 1, we established the baseline decision switching behavior: participants switched more frequently following incorrect trials than correct trials, but there was no significant difference between win and loss trials. In Experiment 2, we highlighted the utility (win or loss) or performance (correct or incorrect) dimension of the *chosen* outcome and we found that the difference in switching frequency was enlarged along the highlighted dimension. However, Experiment 3 showed that when using non-specific saliency emphasis of the outcome, the saliency effect was abolished. We further conducted simultaneous EEG recordings using specific saliency emphasis and found that the feedback-related negativity, P300, and late positive potential could collectively encode saliency modulation of behavioral switching. Lastly, both the frontal and parietal theta-band power encoded the outcome when it was made more salient. Together, our findings suggest that specific outcome saliency can modulate behavioral decision switching between choices and our results have further revealed the neural signatures underlying such saliency modulation. Altering the saliency of an outcome may change how information is weighed during outcome evaluation and thus influence future decisions.

## Introduction

Humans derive pleasure from the utility (win or loss) of an outcome and learn performance accuracy (correct or incorrect) from post-decision feedback. However, this process can be influenced by low-level features of stimuli (e.g., color, contrast, luminance or motion) as well as higher-order concepts (e.g., expressions or warnings)^1–4^. Saliency, which is intimately linked with our motivation and attention^3,5^, refers to aspects of a stimulus that may be arousing^6^ and influence where or to what our attention is directed^7,8^. Several studies have reported that perceptual saliency may interplay with decision making and thus influence subsequent choices. For instance, morality saliency promotes forgiveness and prosocial behavior^9^ and saliency information on health can improve dietary choices^10^. By manipulating relative visual attention or reducing salient information about an investment stock, an individual's investment decisions can be changed^11–13^. Although saliency-based decisions have been widely studied in behavioral science, it is still unclear how our brain represents such saliency signals, which in turn guide subsequent decisions.

Humans have a dedicated neural mechanism to learn from feedback and to switch to a different strategy when outcomes are not as good as expected (e.g., loss in gambling)^14–16^. Previous electroencephalogram (EEG) studies have identified several neural signatures that are sensitive to outcome evaluation including the feedback-related negativity (FRN; also known as medial frontal negativity), the P300, and the late positive potential (LPP). The FRN is a negative deflection at fronto-central recording sites that peaks between 250 and 350 ms after feedback^16–19^. It originates from the medial prefrontal cortex, reflects reward-related outcomes (i.e., utility and performance) or prediction errors^14,15,20,21^, and predicts future decisions^22^. The P300, on the other hand, peaks between 300 to 600 ms after feedback and has the most positive deflection at posterior electrode locations. The P300 often accompanies the FRN^16,23–26^ and is sensitive to various aspects of reward outcome, including the magnitude of reward^24,27^, the valence of a reward^18,25,28^, or both^29,30^. Notably, it also reflects behavioral adjustments based on explicit rules^31^. Lastly, the LPP, which has maximal signals over the anterior frontal or fronto-central sites, has been consistently observed in both perceptual decision making^32–34^ and economic outcome evaluation^35–37^, indicating a possible integrative role in decision making^38^. Together, these event-related potential (ERP) components play an important role in evaluating outcomes and forming decisions. In this study, we further investigated whether these ERP components were modulated by saliency of the outcome and how they collectively encoded saliency-modulated behaviors related to decision making.

In addition to ERPs, neural oscillatory activity (e.g., theta-band power) is also involved in reward processing, in particular for outcome evaluation and response monitoring. On the one hand, enhanced midline-frontal theta oscillations are associated with outcome-related negativity during loss conditions^39,40^, and compared to wins, losses are associated with enhanced power and phase coherence in the theta frequency band^17^. On the other hand, oscillatory activity associated with response error processing shows enhanced theta power following incorrect compared to correct responses^41,42^. Furthermore, feedback anticipation is accompanied by a shift in the power spectrum from relatively lower (delta and theta) to higher (alpha and beta) frequency power, with enhanced power following losses compared to wins in the theta, alpha, and beta frequency bands, but decreased power in the delta frequency band^43^. Given the role of neural oscillations in outcome evaluation and response monitoring, the present study investigated whether neural oscillations encoded saliency modulation of decision switching behavior.

In this study, we conducted three experiments to examine the modulation of behavioral switching in a simple gambling task by manipulating the saliency of the outcome. Briefly, in Experiment 1, we estimated baseline behavioral decision switching using no saliency emphasis on the outcome of the decision. In Experiments 2 and 3, we emphasized either utility (win/loss) or performance (correct/incorrect). Finally, to examine the underlying neural activity of saliency in decision making, EEG recordings were taken during Experiment 2 from a subset of participants.

## Results

### Saliency emphasis modulated behavioral switching

**Figure 1:**
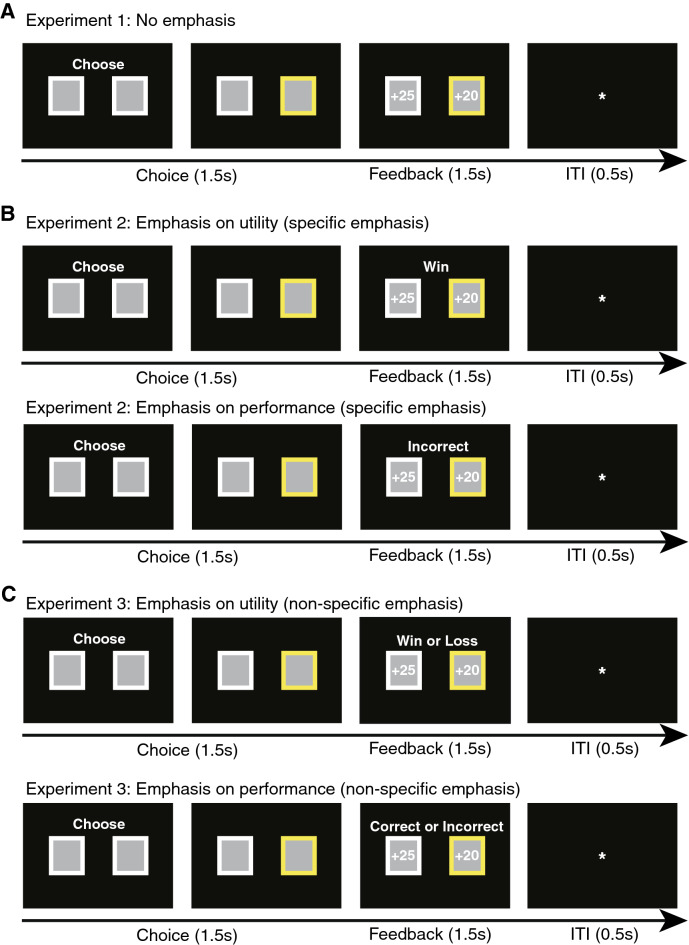
Task. (**A**) Task for Experiment 1. At the beginning of each trial, two gambling cards were presented with the value of the card not shown and participants were required to choose one card within 1.5 s. The chosen card was then highlighted in yellow to indicate the participant’s selection. Then feedback was provided by displaying the outcome associated with the chosen card (still highlighted) and the alternative outcome associated with the unchosen card. This was followed by an inter-trial-interval (0.5 s). There was no saliency emphasis in this experiment. (**B**) Task for Experiment 2. To emphasize the utility (win or loss) or performance (correct or incorrect) dimension of the *chosen* card, a specific highlight message (‘Win’, ‘Loss’, ‘Correct’, ‘Incorrect’) about the chosen outcome was displayed. (**C**) Task for Experiment 3. The procedure was the same as Experiment 2, except that a non-specific highlight about the emphasis dimension (‘Win or Loss’, ‘Correct or Incorrect’) was displayed.

To investigate the impact of feedback and saliency emphasis on decision-making strategies, we employed a behavioral switching task where participants were asked to choose between two gambling cards. Participants received trial-by-trial feedback of the outcome for both their chosen card and the unchosen card (Fig. 1). There were four types of outcomes (Supplementary Table 1): win-correct (WC), win-incorrect (WI), loss-correct (LC), and loss-incorrect (LI). ‘Win’ and ‘loss’ mean that the chosen card yields a reward and penalty, respectively. ‘Correct’ and ‘incorrect’ mean that the chosen card yields a better outcome (a larger reward or a smaller penalty) or a worse outcome (a smaller reward or a larger penalty) compared to the unchosen card, respectively. Based on the feedback received following their decision, participants might adjust their choice in the subsequent trial because previous studies using reinforcement learning have consistently indicated a behavioral tendency of choosing alternative choices following loss or less optimal decisions^22,44,45^. We therefore analyzed the switching frequency of cards (i.e., percentage of trials in which the participant chose a different card in the subsequent trial), which could indicate adjustment of behavior, as a function of the four possible outcomes. Importantly, we tested whether switching frequency could be modulated by emphasizing the saliency of a specific aspect of the outcome. To manipulate saliency, we displayed a text message above the outcome emphasizing either utility (win/loss) or performance (correct/incorrect) during each session (note that all trials of the same session had the same saliency emphasis). Each participant underwent two sessions and the order of saliency emphasis was counterbalanced.

**Figure 2:**
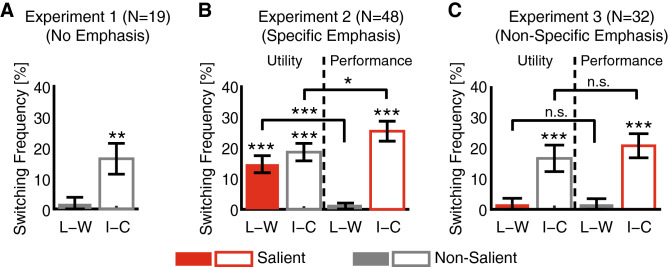
Behavioral results. (**A**) Experiment 1. When there was no saliency emphasis, participants switched more frequently following incorrect trials than correct trials but there was no significant difference between loss and win trials. (**B**) Experiment 2. Specific saliency emphasis increased the difference in switching frequency congruent to the emphasized dimension. (**C**) Experiment 3. Non-specific saliency emphasis did not increase the difference in switching frequency congruent to the emphasized dimension. Error bars denote one SEM across participants. Asterisk indicates a significant difference using two-tailed one-sample t-test: *P < 0.05, **P < 0.01, and ***P < 0.001. n.s.: not significant. Red: congruent/salient. Gray: incongruent/non-salient. Solid bars denote loss–win whereas open bars denote incorrect–correct.

We first established the baseline performance in Experiment 1 (Fig. 2A; see Supplementary Fig. 1A for absolute switching frequencies for each condition), where we did not have any saliency emphasis. Participants switched more frequently following incorrect trials than correct trials (two-tailed paired t-test: *t*(18) = 3.25, *P* = 0.0044, Cohen’s *d* = 0.77). However, there was no significant difference between loss trials and win trials (*t*(18) = 0.80, *P* = 0.43, *d* = 0.19).

We next included saliency modulation in Experiment 2 (Fig. 2B, Supplementary Fig. 1B). The difference in switching frequency following loss versus win (L–W) trials was congruent with the emphasis on utility and was thus salient when utility was emphasized. Indeed, participants switched more frequently following loss trials than win trials when utility was emphasized (*t*(47) = 5.28, *P* = 3.21 × 10^−6^, *d* = 0.77). However, this was not the case when performance was emphasized (*t*(47) = 1.11, *P* = 0.27, *d* = 0.16), suggesting that emphasis on utility specifically increased switching frequency following loss trials (*t*(47) = 4.63, *P* = 2.88 × 10^−5^, *d* = 0.67). This was further confirmed by a significant interaction between saliency emphasis and utility (*F*(1,47) = 21.5, *P* = 2.88 × 10^−5^, η_p_^2^ = 0.31).

On the other hand, the difference in switching frequency following incorrect versus correct (I–C) trials was congruent with the emphasis on performance and was thus salient when performance was emphasized. A significant interaction between saliency and performance was also observed (*F*(1,47) = 5.42, *P* = 0.024, η_p_^2^ = 0.10): although participants switched more frequently following incorrect trials than correct trials when either utility (*t*(47) = 6.53, *P* = 4.27 × 10^−8^, *d* = 0.95) or performance (*t*(47) = 7.86, *P* = 4.08 × 10^−10^, *d* = 1.14) was emphasized, the difference was greater when performance was emphasized (*t*(47) = − 2.33, *P* = 0.024, *d* = 0.34). Together, our results suggest that saliency emphasis can increase the difference for the congruent (thus salient) task aspect. Therefore, adjustment of behavior (shown in switching frequency) can be modulated by saliency emphasis.

### Non-specific saliency emphasis did not modulate behavioral switching

To test whether saliency emphasis had to be specific to the chosen outcome, we conducted Experiment 3 with non-specific saliency emphasis—only the dimension of the emphasis (utility or performance) was shown to participants (Fig. 1C), but not the specific emphasis on the chosen outcome. Note that all trials in the same session had the same saliency emphasis. Here, we found that saliency modulation was abolished (Fig. 2C, Supplementary Fig. 1C): unlike the outcome in Experiment 2, we found no significant difference in switching frequency between loss and win trials when either utility (*t*(31) = 0.85, *P* = 0.40, *d* = 0.15) or performance (*t*(31) = 0.92, *P* = 0.37, *d* = 0.17) was emphasized, and we found no significant difference between the two emphases (*t*(31) = 0.001, *P* = 0.999, *d* < 0.001), a result similar to Experiment 1 (Fig. 2A) in which no emphasis was indicated. On the other hand, although participants switched more frequently after incorrect trials than correct trials when either utility (*t*(31) = 3.85, *P* = 5.53 × 10^−4^, *d* = 0.69) or performance (*t*(31) = 5.28, *P* = 9.62 × 10^−6^, *d* = 0.95) was emphasized, no significant difference was found between the two saliency emphases (*t*(31) = − 1.18, *P* = 0.25, *d* = 0.21). We further confirmed that there were no significant interactions between saliency and utility (*F*(1,31) < 0.001, *P* = 0.999, η_p_^2^ < 0.001) or between saliency and performance (*F*(1,31) = 1.40, *P* = 0.245, η_p_^2^ = 0.04). Together, our results suggest that non-specific saliency emphasis did not modulate behavioral switching.

In addition to behavioral switching, we also investigated subjective ratings of outcomes in a subset of participants (18 participants from Experiment 2 and 18 participants from Experiment 3 provided ratings) and measured ratings of surprise (i.e., how surprised participants felt for the outcome) using an 11-point analogue Likert scale (0 = not at all, 10 = very intensely) for each experimental condition. We found no significant difference in surprise ratings between Experiments 2 and 3 (two-tailed unpaired t-test: *t*(34) = 1.73, *P* = 0.092); and within each experiment, we found no significant main effects or interactions for surprise ratings (three-way repeated-measure ANOVA of saliency × utility × performance: all Ps > 0.05) except for utility × performance in Experiment 3 (*P* < 0.05). Therefore, self-reported surprise confirmed that behavioral adjustments under different conditions were not based on different anticipation of outcome.

### The FRN, P300 and LPP collectively encoded saliency modulation of behavioral switching

**Figure 3:**
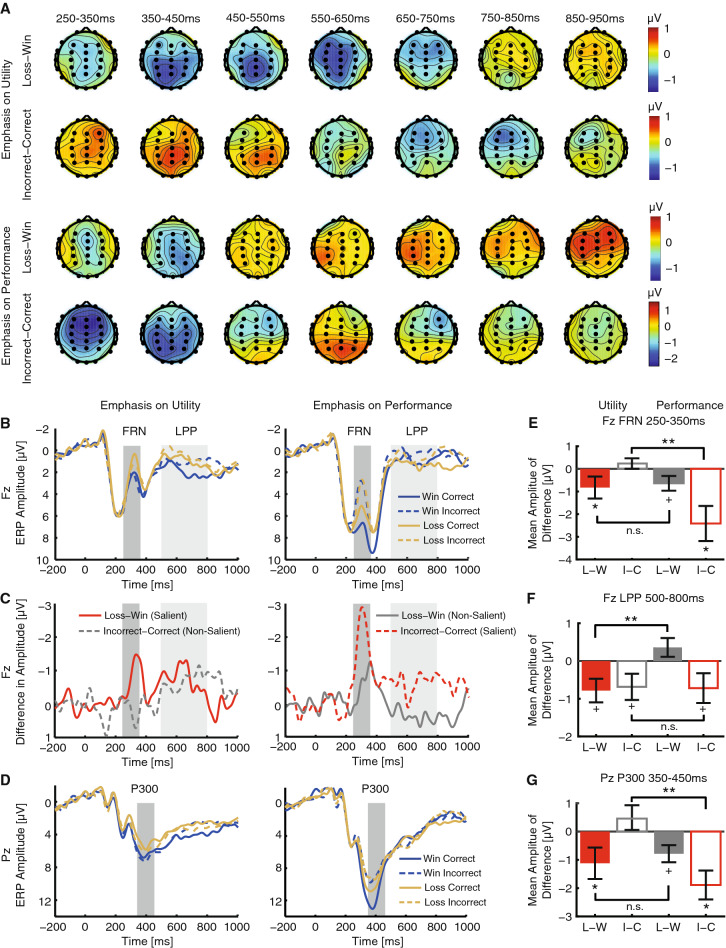
ERP results from Experiment 2. (**A**) The scalp topography of the difference waveform between loss and win trials, and between incorrect and correct trials. (**B**) ERP at electrode Fz. Gray shaded areas denote the FRN (250–350 ms) and LPP (500–800 ms) intervals. Blue: win. Yellow: loss. Solid line: correct. Dashed line: incorrect. (**C**) Difference waveforms. Red: congruent/salient. Gray: incongruent/non-salient. Solid lines denote loss–win whereas dashed lines denote incorrect–correct. (**D**) ERP at electrode Pz. Gray shaded area denotes the P300 (350–450 ms) interval. (**E**) Mean amplitude of difference for the FRN. (**F**) Mean amplitude of difference for the LPP. (**G**) Mean amplitude of difference for the P300. Error bars denote one SEM across participants. Asterisk indicates a significant difference using two-tailed one-sample t-test: ^+^*P* < 0.1, *P < 0.05, and **P < 0.01. n.s.: not significant. Red: congruent/salient. Gray: incongruent/non-salient. Solid bars denote loss–win whereas open bars denote incorrect–correct. ERP, event-related potential;  FRN, feedback-related negativity; LPP, late positive potential.

To further investigate the neural signatures of saliency modulation of behavior, we examined three ERP components: the FRN, P300, and LPP, which have been indicated in feedback processing and outcome evaluation (see “Introduction” for details). In Experiment 2, 18 participants underwent EEG recording during the gambling task. We confirmed that these participants had no significant difference in behavior compared with the rest of the participants in Experiment 2 (N = 30; two-tailed unpaired t-test for each condition: all *P* values > 0.09; four-way ANOVA of participant group X saliency X utility X performance: main effect of participant group and interactions with participant group: all *P* values > 0.19). The scalp topography of the difference waveform of L–W and I–C (Fig. 3A) showed a pronounced activity at Fz for 250–350 ms (FRN; see also Supplementary Fig. 2A for scalp topographies around FRN peak), and Pz for 350–450 ms (P300) and 500–800 ms (LPP); therefore we focused on these components. As expected, we observed clear FRN, LPP, and P300 components after onset of the feedback message for four types of feedback (Fig. 3B–D).

First, the FRN showed a significant interaction between saliency and performance (Fig. 3B,C,E; *F*(1,17) = 10.01, *P* = 0.0057, η_p_^2^ = 0.37): when performance was emphasized, the FRN was more negative in incorrect trials than in correct trials (Fig. 3E; two-tailed paired t-test, *t*(17) = − 3.12, *P* = 0.0062, *d* = − 0.76), but not when utility was emphasized (*t*(17) = 1.03, *P* = 0.32, *d* = 0.25), suggesting that saliency emphasis of performance modulated FRN activities. However, no significant interaction between saliency and utility was observed (*F*(1,17) = 0.09, *P* = 0.76, η_p_^2^ = 0.005): although the FRN was more negative in loss trials than in win trials when either utility or performance was emphasized, there was no significant difference between the two emphases (*t*(17) = − 0.30, *P* = 0.76, *d* = − 0.07), suggesting that saliency emphasis of utility did not modulate FRN activities.

Second, the P300 mirrored FRN results (Fig. 3D,G): the P300 was more positive in correct trials than in incorrect trials when performance was emphasized (*t*(17) = 3.71, *P* = 0.0017, *d* = 0.90) but not when utility was emphasized (*t*(17) = − 1.13, *P* = 0.28, *d* = 0.27), resulting in a significant interaction between saliency and performance (*F*(1,17) = 9.01, *P* = 0.0080, η_p_^2^ = 0.35). However, the P300 showed no significant interaction between saliency and utility (*F*(1,17) = 0.28, *P* = 0.60, η_p_^2^ = 0.017).

In contrast, the LPP showed a significant interaction between saliency and utility (Fig. 3B,C,F; *F*(1,17) = 8.64, *P* = 0.0092, η_p_^2^ = 0.337): the LPP was more enhanced in win trials than loss trials when utility was emphasized (*t*(17) = 1.81, *P* = 0.088, *d* = 0.44) but not when performance was emphasized (*t*(17) = − 1.45, *P* = 0.17, *d* = 0.35), suggesting that saliency emphasis of utility modulated LPP activities, an effect that was not found in the FRN or P300. On the other hand, no significant interaction between saliency and performance was found (*F*(1,17) = 0.35, *P* = 0.56, η_p_^2^ = 0.02). Although the LPP was more positive in correct trials than in incorrect trials when either utility or performance was emphasized, there was no significant difference between the two emphases (*t*(17) = − 0.007, *P* = 0.994, *d* = 0.002), suggesting that saliency emphasis of performance did not modulate LPP activities.

Lastly, similar results were found when the FRN and LPP were evaluated at FCz (Supplementary Fig. 2B–E). Furthermore, our results were qualitatively the same when using peak amplitude of the FRN, P300 and LPP, as well as mean amplitude of 5 channels (Fz, FCz, F3, F4, Cz) around the frontal areas for the FRN and LPP and 4 channels (Pz, CPz, P3, P4) around parietal areas for the P300. However, the peak latency of these components did not encode saliency modulation.

Together, the FRN, P300 and LPP collectively encode saliency modulation: the FRN and P300 encode emphasis on performance whereas the LPP encodes emphasis on utility. Given the longer latency of the LPP time window compared with that of the FRN and P300, it suggests that modulation of utility is processed later compared with modulation of performance.

### The frontal and parietal theta-band power encoded the salient aspect of outcome

**Figure 4:**
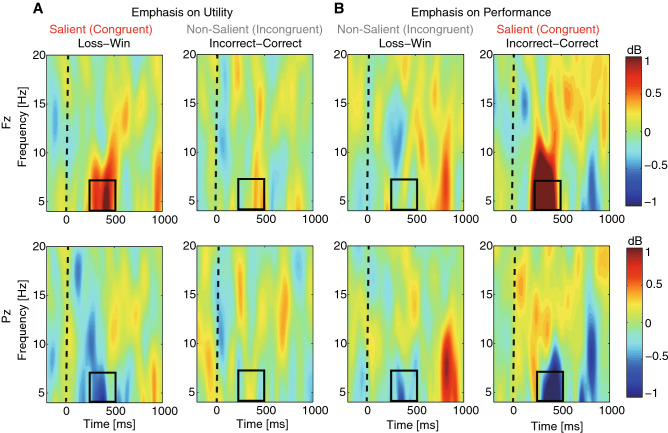
Results for time–frequency analysis from Experiment 2. Time–frequency plots depicting the power of induced oscillations for the difference of outcomes (loss–win, incorrect–correct) at electrodes Fz and Pz. (**A**) Emphasis on utility. (**B**) Emphasis on performance. The theta (4–7 Hz) band power encoded emphasized outcomes.

To investigate whether neural oscillations, in addition to ERP, also encoded saliency modulation of behavior, we next conducted a time–frequency analysis based on three frequency bands: theta (4–7 Hz), alpha (8–12 Hz), and beta (13–20 Hz), during time intervals of 250–500 ms and 700–900 ms (Fig. 4). In particular, the scalp topography of saliency modulation showed a pronounced theta-band power at Fz and Pz for 250–500 ms (Supplementary Fig. 3).

During time interval 250–500 ms, we observed significant interactions in Fz theta-band power not only between saliency and utility (*F*(1,17) = 12.8, *P* = 0.0023, η_p_^2^ = 0.43) but also between saliency and performance (*F*(1,17) = 14.4, *P* = 0.0014, η_p_^2^ = 0.46). In particular, the theta-band power was enhanced for loss trials compared with win trials when utility was emphasized (Fig. 4A; two-tailed paired t-test, *t*(17) = − 3.73, *P* = 0.0017, *d* = − 0.90) but not when performance was emphasized (*t*(17) = 0.51, *P* = 0.617, *d* = 0.12). In contrast, the theta-band power was enhanced for incorrect trials compared with correct trials when performance was emphasized (Fig. 4B; *t*(17) = − 5.39, *P* = 4.9 × 10^−5^, *d* = − 1.31) but not when utility was emphasized (*t*(17) = 0.81, *P* = 0.43, *d* = 0.20). Together, our results suggest that saliency modulates theta-band power for both utility and performance.

Mirroring this result, reduced (but not enhanced) Pz theta oscillations were found for both saliency emphases (Fig. 4), suggesting that the parietal theta-band power could also encode saliency modulation. However, we found no saliency modulation of theta-band power during the time interval 700–900 ms nor any saliency modulation of alpha-band or beta-band power.

Together, these results suggest that in addition to ERPs’ collective coding of saliency modulation, theta-band power can encode both saliency emphases on utility and performance.

### Single-trial analysis revealed association between neural activity and behavior

We have shown above that saliency could modulate behavioral switching and neural representation of feedback. We lastly confirmed that the neural signatures for saliency and feedback were associated with switching behavior. We constructed a generalized linear mixed model using single-trial ERPs or oscillations to predict subsequent behavioral switching. First, we found a significant interaction between the FRN, LPP, P300, and saliency emphasis in the full model (slope; β = − 0.00012, *P* = 0.002). We then applied likelihood ratio tests to compare the full model with the fixed effects of the FRN, LPP, and P300 (and separately for theta oscillations) with a reduced model lacking one individual fixed effect. Indeed, we found that the FRN (χ^2^(17) = 15.54, *P* = 0.04), LPP (χ^2^(17) = 17.72, *P* = 0.02), and theta-band power (250–500 ms after feedback onset; Fig. 4; χ^2^(5) = 7.24, *P* = 0.027) significantly contributed to predict single-trial behavioral switching. Although the P300 did not contribute to predict single-trial behavioral switching (χ^2^(17) = 6.36, *P* = 0.61), it contributed to predict the absolute value of outcomes (χ^2^(18) = 15.54, *P* = 0.04). Together, our single-trial analysis suggests that the neural signatures indexing saliency modulation of feedback are coupled with behavioral switching.

## Discussion

We investigated saliency modulation of behavioral switching in a simple gambling task in which either the utility (win/lose) or performance (correct/incorrect) aspect of the outcome was emphasized. Behaviorally, when there was no emphasis on saliency (Experiment 1), participants switched more frequently following incorrect trials than correct trials, but there was no significant difference between win and loss trials. Experiment 2 showed that when there was specific saliency emphasis of the chosen outcome, participants switched more frequently along the emphasized dimension (utility or performance). However, this effect was abolished when using non-specific saliency emphasis of the outcome (Experiment 3). Neurally, the EEG data revealed that the FRN, P300, and LPP collectively encoded the emphasized dimension. Lastly, both the frontal and parietal theta-band power encoded the saliency modulation of behavioral switching.

In this study, we found that participants switched more frequently following incorrect trials than correct trials regardless of saliency emphasis whereas participants only switched more frequently following loss trials than win trials when utility (win/loss) was specifically emphasized (Fig. 2). It is also worth noting that the P300 was significantly larger when performance (correct/incorrect) was emphasized than when utility was emphasized (two-tailed paired t-test: *t*(17) = 3.52, *P* = 0.003, *d* = 0.85), likely because participants could relate the performance feedback rather than the utility feedback to their preceding act of choosing, a result that is consistent with prior reports showing that the P300 is larger in events that have to be acted on compared to those that are independent of the participant’s response^46^. Therefore, our results indicate that different psychological processes may underlie response to performance feedback versus utility feedback, which is in turn supported by separate neural mechanisms (Fig. 3; note that each neural signature only encoded one saliency emphasis). Computationally, behavioral switching may be achieved by On and Off switch neurons^47^. In non-human primates, behavioral switching of win-stay/lose-switch has been attributed to activity in the posterior lateral orbitofrontal cortex (lOFC) and amygdala, and interestingly, lOFC-amygdala connectivity can be modulated dynamically by the relevance of reward information^48^. A future extension of our study will be to understand the neural computations underlying behavioral switching as well as to identify the sources of the neural signatures of saliency modulation and the functional connectivity between these sources.

In an early seminal study^14^, the FRN is shown to be sensitive to the utility (gain/loss) of the chosen outcome but not the performance (correctness) of choices. However, this idea has been challenged by later studies showing that the amplitude of the FRN regarding the utility or performance dimension of the feedback depends on which dimension is more salient^49^, which is consistent with our present findings. Here, we provide new data relative to this literature. First, we characterized not only the FRN, but also the behavior, which is indexed by the frequency of switching to alternative options in subsequent trials. Second, we not only quantified the baseline switching behavior but also found that only specific saliency emphasis could modulate behavioral switching. Third, we found that the FRN only encoded the saliency emphasis on performance but not utility. Fourth, we showed that different ERP components could encode saliency emphasis on utility, and our data indicated a latency difference (i.e., the earlier ERP component encoded emphasis on performance while the later ERP component encoded emphasis on utility). Lastly, our task used an explicit semantic saliency emphasis rather than an implicit emphasis such as color^14^ or a combination of color and symbol^49^. Since EEG data in this study was only collected in Experiment 2 in which saliency was manipulated, future experiments will be needed to characterize the FRN profile during the baseline condition (Experiment 1) as well as during the non-specific emphasis condition (Experiment 3).

Two different theories have been proposed to illustrate the role of the FRN in outcome evaluation^20,50–52^. According to the reinforcement learning theory, saliency may unconsciously drive preferential attention towards outcomes with certain information, whereas according to the motivational account, saliency may enlarge the affective significance of the outcome. Both theories indicate a modulatory effect of saliency on the FRN when representing utility or performance information. In this study, we found that three ERP components (the FRN, P300, and LPP) collectively encoded saliency modulation of behavior, presumably by amplifying the attentional or motivational responses toward those outcomes. Consistent with the present result, in an instrumental learning task, a prior study showed that the FRN encoded the prediction error signal whereas the P300 was predictive of the decision on the next trial, which directly established the link between cortical feedback processing and consecutive behavioral switches^53^. Although our present results could not distinguish between the roles of attention and motivation, future studies are needed to disentangle the contribution of attention and motivation in saliency-based decision making.

We found that the LPP encoded saliency emphasis of utility in a relatively late time window (compared with the FRN and P300). This is consistent with the LPP’s role in accumulating evidence in decision making^32^. In addition, single-neuron experiments on primates show that visual saliency and value information are processed with different delays, with shorter latency for saliency but longer latency for value information^3^, consistent with our finding that utility information was processed in a later time window. It is worth noting that in the present study we analyzed LPP at the electrode Fz instead of its conventional site Pz^32–34^. The choice of Fz LPP was based on the scalp topography of the difference waveform and because Pz LPP did not demonstrate saliency modulation (Fig. 3D). The Fz LPP still showed a positive deflection although the amplitude was relatively smaller compared with Pz LPP. Similar patterns of the Fz LPP can also be observed in other reports with analysis from both frontal and parietal regions^54–56^.

Consistent with the findings in our present study, neural oscillatory activity such as theta-band oscillation has previously been shown to accompany ERPs during outcome evaluation^39,41,57^. For example, the error related negativity (ERN) is dominated by partial phase-locking of intermittent theta-band EEG activity^57^, and losses (compared to wins) are associated with enhanced power^17,40^ and phase coherence^17^ in the theta frequency band. Furthermore, prior studies indicate that theta power is enhanced following incorrect compared to correct responses^41,42^. In the present study, we found that both frontal and parietal theta power was modulated by outcome saliency. The frontal and parietal theta power may originate from the cingulate gyrus^34^, which may in turn stem from the hippocampus through hippocampal projections to the cingulate gyrus^58,59^. Such hippocampal-cingulate projections have been supported by increasingly synchronized unit activity in theta bursts as an animal prepares to make a learned response^58,59^.

Saliency can interplay with value computation in decision making. It has been shown that perceptual saliency competes with expected value, and both influence the saccadic end point within an object^3,60,61^. Manipulating the relative amount of visual attention between two alternative options can influence subsequent choices^11^. Consistent with our present findings, behavioral economics studies show that deemphasizing the saliency of purchase price of an investment stock substantially reduces the investor’s propensity to sell risky assets with capital gains^12^. On the other hand, reward biases visual saliency and subsequent strategies^62^. Interestingly, reward can create oculomotor saliency—a reward-associated distractor can change saccade trajectories even when participants expect the distractor and try to ignore it^63^. Our results have further extended these previous studies by showing that outcome saliency, even when it is redundant in nature, has an impact on subsequent decisions. We have further shown specificity of saliency modulation: only when saliency emphasis is specific to the chosen outcome, does it exert an influence on subsequent choices.

In conclusion, the present study has identified neural signatures of saliency modulation of behavior. Our findings suggest that the reward system is orchestrated by saliency and underscores the key role of specific highlights in modulating goal-directed behavior. Such saliency modulation can be utilized in real life situations, such as product marketing, policy making, and even casino gambling to influence individual choices.

## Methods

We conducted three experiments. In Experiment 1, we established the baseline behavior by using no saliency emphasis on the outcome of gambling decisions (Fig. 1A). In Experiment 2, we used highlights that not only indicated the emphasis dimension but also were specific to the chosen outcome (Fig. 1B). In Experiment 3, highlights only indicated the emphasis dimension but were not specific to the chosen outcome (Fig. 1C). To investigate the neural signatures of saliency modulation, a subset of participants in Experiment 2 had simultaneous EEG recording.

### Participants

Nineteen healthy, right-handed participants (7 male; mean age ± SD: 21.54 ± 1.56 years) participated in Experiment 1, 48 separate healthy, right-handed participants (20 male; 21.84 ± 2.23 years) participated in Experiment 2, and 32 separate healthy, right-handed participants (16 male; 21.26 ± 2.81 years) participated in Experiment 3. Eighteen participants from Experiment 2 had simultaneous EEG recording. All participants had normal or corrected-to-normal vision, and no neurological or psychiatric disorders. All participants provided written informed consent according to protocols approved by the South China Normal University Institutional Review Board, and all methods were carried out in accordance with the approved guidelines.

### Stimuli and procedure

Participants were seated comfortably about 1.1 m in front of a computer screen in a dimly lit and electromagnetically shielded room. Experiments were administered on a 19-inch (37.7 × 30.1 cm) IBM LCD display (1,280 × 1,024 screen resolution). We used E-prime (Psychology Software Tools, Inc. Pittsburgh, PA, USA, www.pstnet.com/e-prime) for stimulus presentation and response recording. At the beginning of each trial, participants viewed two gambling cards and were required to choose one card by pressing the corresponding button within 1.5 s (Fig. 1). The trial was discarded if participants did not make a response within 1.5 s. The chosen card was highlighted by a yellow box immediately after button press for the rest of the 1.5 s. Subsequently, the outcome associated with both the chosen card and the unchosen card was shown for 1.5 s, followed by an inter-trial-interval (ITI) of 0.5 s.

There were four types of outcomes: win-correct (WC), win-incorrect (WI), loss-correct (LC), and loss-incorrect (LI). ‘Win’ and ‘loss’ mean that the chosen card yields a reward or penalty, respectively (Fig. 1). ‘Correct’ and ‘incorrect’ mean that the chosen card yields a better outcome (a larger reward or a smaller penalty) or a worse outcome (a smaller reward or a larger penalty) outcome compared to the unchosen card, respectively. Four corresponding examples were given and explicitly explained to each participant (see Fig. 1 for examples). Unbeknownst to participants, all outcomes were predetermined (the same for all participants) and pseudo-randomized across conditions (see Supplementary Table 1 for the complete list of outcomes; note that there was an equal number of trials for each type of outcome). Specifically, the value of the chosen card was randomly decided (in integers) from a uniform distribution ranging from − ¥40 to + ¥40 (~ $6), whereas the value of the unchosen card was also determined randomly from a uniform distribution, but with the constraint that the difference between the chosen and unchosen outcomes was less than ¥20 (but no less than ¥2). Note that it was possible that the chosen and unchosen cards had an opposite sign (Supplementary Table 1; 11/160 pairs). However, we found qualitatively the same results when we excluded the pairs of outcomes with an opposite sign. The values of the chosen and unchosen cards were independent of card positions. Participants were told that their goal was to earn as much money as possible, and they were free to employ any strategies to achieve that goal.

Before the experiment, participants were informed that one trial would be selected randomly from the experiment for payment. Ten practice trials were given before the experiment, allowing participants to get familiar with the procedure. No reward was given for practice trials.

### Saliency manipulation

Each participant underwent two sessions in one of three experiments. In Experiment 1, both sessions were the same and had no saliency emphasis (Fig. 1A). In Experiments 2 and 3, each session had a different saliency emphasis (emphasizing either utility or performance for all trials within the same session). We used a block design to avoid trial-by-trial variation in saliency emphasis and thus achieve equal predictability of saliency emphasis across experiments. To emphasize utility (win/loss) or performance (correct/incorrect), a highlight message was displayed above the outcomes (Fig. 1B,C). In Experiment 2, the highlight was specific to the outcome, whereas in Experiment 3, the highlight only indicated the emphasis dimension (i.e., “Win or Loss” for emphasis on utility and “Correct or Incorrect” for emphasis on performance) but was not specific to the chosen outcome. Thus, in contrast to Experiment 2, the highlight in Experiment 3 only reminded participants which dimension they should focus on without providing any specific information about the chosen outcome. Each session consisted of 2 blocks of 80 trials each, and there was a short break between the two blocks. For Experiments 2 and 3, the two sessions were counterbalanced across participants.

It is worth noting that in Experiments 2 and 3, one of the task aspects (loss–win or incorrect–correct) became congruent with the emphasized dimension (utility or performance) and thus became salient after emphasis. Specifically, the difference in switching frequency following loss versus win (L–W) trials was congruent with the emphasis on utility and was thus salient when utility was emphasized. Similarly, the difference in switching frequency following incorrect versus correct (I–C) trials was congruent with the emphasis on performance and was thus salient when performance was emphasized.

### Electroencephalogram (EEG): data recording

EEGs were recorded using a digital AC amplifier from 32 scalp sites with tin electrodes mounted in an elastic cap (NeuroScan4.5) according to the International 10–20 system. EEGs were recorded from the following sites: frontal: Fp1, Fp2, F7, F3, Fz, F4, F8; fronto-central: FC3, FCz, FC4; central: C3, Cz, C4; central-parietal: CP3, CPz, CP4; parietal: P7, P3, Pz, P4, P8; fronto-temporal-parietal: FT7, TP7, T7, T8, TP8, FT8; and occipital: O1, Oz, O2. The vertical-oculograms (VEOG) were recorded from left supra-orbital and infra-orbital electrodes. The horizontal electro-oculograms (HEOG) were measured from electrodes placed lateral to the outer canthi of the eyes. The ground electrode was placed on the forehead. One reference electrode was placed at the left mastoid and the other at the right mastoid, and all recordings were referenced to the right mastoid. All impedance were maintained below 5 kΩ. EEG and electro-oculogram (EOG) were amplified using a 0.05–70 Hz band-pass filter and were continuously sampled at 500 Hz.

### EEG: data preprocessing

EEG data were processed using EEGLAB^64^, an open source toolbox running in the MATLAB environment, and in-house MATLAB functions. The continuous EEG data were re-referenced to the average of the left and right external mastoid signals to avoid biasing the data towards one hemisphere^65,66^. The data were filtered using a digital zero-phase shift band-pass filter of 0.5–20 Hz with a slope of 24 dB/octave. Then continuous EEG data were epoched into 2-s segments (–500 to 1,500 ms relative to outcome onset), and the pre-stimulus interval (− 200 to 0 ms) was used as the baseline. The data were then baseline corrected by subtracting the average activity during the baseline period. Eye movements or blinks were identified and removed using an independent component analysis (ICA) algorithm. In all datasets, these independent components had a large EOG channel contribution and a frontal scalp distribution. Trials contaminated by any remaining eye movement or blinks were rejected by visual inspection. Bad channels were interpolated using the average voltage from their surrounding electrodes. Consequently, 8.06 ± 13.8% (mean ± SD) of trials were discarded from further analysis. Importantly, after excluding trials due to artifacts, the average number of valid trials were similar at each condition (three-way repeated-measure ANOVA, all *F*(1,17) < 1, all *P*s > 0.05) (Emphasis on utility: WC: 36.72 ± 5.20 (mean ± SD), WI: 36.83 ± 4.59, LC: 36.61 ± 6.34, LI: 37.22 ± 4.17; Emphasis on performance: WC: 36.77 ± 6.16; WI: 36.55 ± 6.40; LC: 36.78 ± 6.45; LI: 36.72 ± 6.23; respectively).

### EEG: event-related potential (ERP) analysis

We first analyzed the ERP components during outcome evaluation. Within each participant, we computed the mean waveform of each type of outcome, time-locked to the onset of the feedback. Single-participant mean waveforms were subsequently averaged to obtain group-level mean waveforms. Consistent with previous studies, we calculated the mean FRN amplitude in the time window of 250 to 350 ms after feedback onset at electrode Fz^14,15^, the mean P300 amplitude in the time window of 350 to 450 ms after feedback onset at electrode Pz^55^, as well as the mean LPP amplitude in the time window of 500 to 800 ms after feedback onset at electrode Fz^35,67^.

### EEG: time–frequency analysis

Besides evoked potentials, phase and non-phase locked neural oscillations are also involved in outcome evaluation. We used an epoch window of − 500 to 1,500 ms relative to outcome onset for frequency analysis. Trial-by-trial time–frequency decomposition was performed using a windowed Fourier transform (WFT) with a fixed 250 ms width Hanning window. The WFT computes a complex time–frequency value F(t,f) at each point (t,f) on the time–frequency plane, extending from − 500 to 1,500 ms (in steps of 2 ms) in the time domain, and from 4 to 20 Hz (in steps of 1 Hz) in the frequency domain. We used a common baseline (starting from − 400 to − 200 ms before outcome onset) for all conditions. This baseline period differed from that used for ERP analysis to avoid the edge effects. Then, the spectra were 10 * log10-transformed (the unit of the log-transformed spectrum is decibel [dB]). For each frequency, the corresponding average baseline spectral power was divided, yielding the baseline-normalized event-related spectral perturbation (ERSP), an estimate of event-related changes in the power spectrum. Then ERSPs were averaged separately for each condition, and plotted at all frequencies, at time points from 200 ms before outcome onset to 1,000 ms after outcome onset. In this study, we focused on the theta (4–7 Hz), alpha (8–12 Hz), and beta (13–20 Hz) frequency bands at two different time intervals (250–500 ms and 700–900 ms). We selected these frequency bands based on previous EEG studies on decision making, in which strong oscillatory activity was observed^43^.

### Statistics

A three-way repeated-measure ANOVA (saliency [utility vs. performance] × utility [win vs. loss trials] × performance [correct vs. incorrect trials]) was performed. The dependent variables were switching frequency, ERP amplitude, or oscillation power. The Mauchly test was used to assess the validity of the sphericity assumption in the ANOVAs. Greenhouse–Geisser corrections were used when sphericity was violated.

## Supplementary information

Supplementary information
