# Supplementary material for: Outcome saliency modulates behavioral decision switching
Source: Sci Rep. 2020 Aug 31;10:14288. doi: 10.1038/s41598-020-71182-9 (PMC7459124; doi:10.1038/s41598-020-71182-9)
Supplement: Supplementary file 1 — Supplementary information [file 41598_2020_71182_MOESM1_ESM.pdf]

Supplementary Information

Outcome saliency modulates behavioral decision switching

Sai Sun[1], Rongjun Yu[2*], Shuo Wang[3*]

[1] Center for Studies of Psychological Application, Key Laboratory of Mental Health and Cognitive Science of Guangdong Province, School of Psychology, South China Normal University, Guangzhou 510631, China.

[2] Department of Psychology, National University of Singapore, Singapore 117570, Singapore

[3] Department of Chemical and Biomedical Engineering and Rockefeller Neuroscience Institute, West Virginia University, Morgantown, WV 26506, USA

Supplementary Table 1. The full list of all pairs of outcomes used in this study that were predetermined.

	Chosen	Unchosen	Difference		Chosen	Unchosen	Difference		Chosen	Unchosen	Difference		Chosen	Unchosen	Difference
**win−correct**	6	3	3	**win−incorrect**	6	22	−16	**loss−correct**	−40	−53	13	**loss−incorrect**	−40	−32	−8
	6	−6	12		7	26	−19		−38	−53	15		−39	−29	−10
	6	−8	14		8	24	−16		−37	−40	3		−39	−25	−14
	7	−5	12		9	12	−3		−36	−39	3		−39	−28	−11
	10	−6	16		10	14	−4		−33	−37	4		−38	−19	−19
	10	6	4		11	29	−18		−32	−40	8		−38	−27	−11
	13	4	9		12	15	−3		−32	−44	12		−36	−23	−13
	14	4	10		15	22	−7		−31	−46	15		−35	−23	−12
	15	4	11		15	21	−6		−31	−38	7		−34	−15	−19
	16	7	9		15	29	−14		−29	−40	11		−34	−21	−13
	17	11	6		16	30	−14		−28	−45	17		−33	−27	−6
	17	6	11		16	23	−7		−28	−42	14		−33	−13	−20
	20	17	3		17	35	−18		−26	−30	4		−32	−22	−10
	21	8	13		20	28	−8		−26	−40	14		−32	−19	−13
	21	5	16		22	38	−16		−25	−36	11		−32	−24	−8
	23	12	11		22	34	−12		−23	−29	6		−31	−23	−8
	23	5	18		22	35	−13		−22	−32	10		−29	−16	−13
	24	21	3		23	39	−16		−22	−28	6		−29	−22	−7
	24	8	16		24	43	−19		−20	−31	11		−29	−26	−3
	25	18	7		24	38	−14		−20	−23	3		−27	−17	−10
	25	15	10		24	38	−14		−19	−34	15		−27	−16	−11
	26	15	11		25	36	−11		−19	−23	4		−26	−18	−8
	27	19	8		27	42	−15		−18	−26	8		−25	−9	−16
	28	25	3		27	37	−10		−18	−25	7		−24	−16	−8
	30	25	5		27	43	−16		−18	−20	2		−24	−17	−7
	30	12	18		28	41	−13		−18	−33	15		−22	−19	−3
	30	22	8		29	48	−19		−17	−21	4		−20	−8	−12
	31	20	11		30	40	−10		−16	−28	12		−14	2	−16
	32	14	18		30	35	−5		−16	−21	5		−13	−2	−11
	33	22	11		32	46	−14		−15	−26	11		−10	−7	−3
	33	29	4		33	39	−6		−15	−23	8		−9	−1	−8
	34	28	6		33	37	−4		−15	−17	2		−9	−5	−4
	35	24	11		34	51	−17		−14	−22	8		−9	−4	−5
	35	27	8		34	41	−7		−12	−20	8		−9	−2	−7
	36	31	5		35	42	−7		−11	−27	16		−8	4	−12
	37	23	14		36	55	−19		−10	−25	15		−8	10	−18
	37	30	7		36	53	−17		−9	−24	15		−7	1	−8
	38	30	8		36	53	−17		−9	−11	2		−7	11	−18
	38	27	11		38	58	−20		−8	−22	14		−6	10	−16
	38	21	17		40	45	−5		−8	−11	3		−6	9	−15

Supplementary Fig. 1

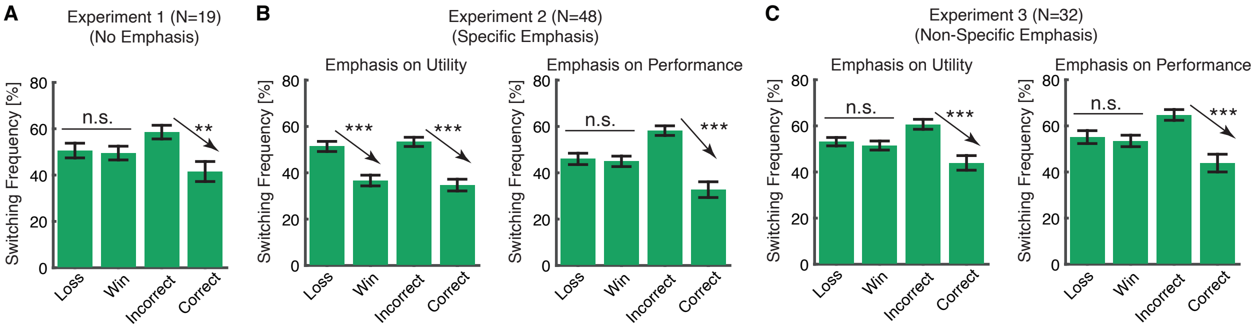

Supplementary Fig.1. Absolute switching frequencies. **(A)** Experiment 1. **(B)** Experiment 2. **(C)** Experiment 3. Error bars denote ±SEM across participants. Asterisks indicate a significant difference between conditions using two-tailed paired t-test: **: P<0.01 and ***: P<0.001. n.s.: not significant.

Supplementary Fig. 2

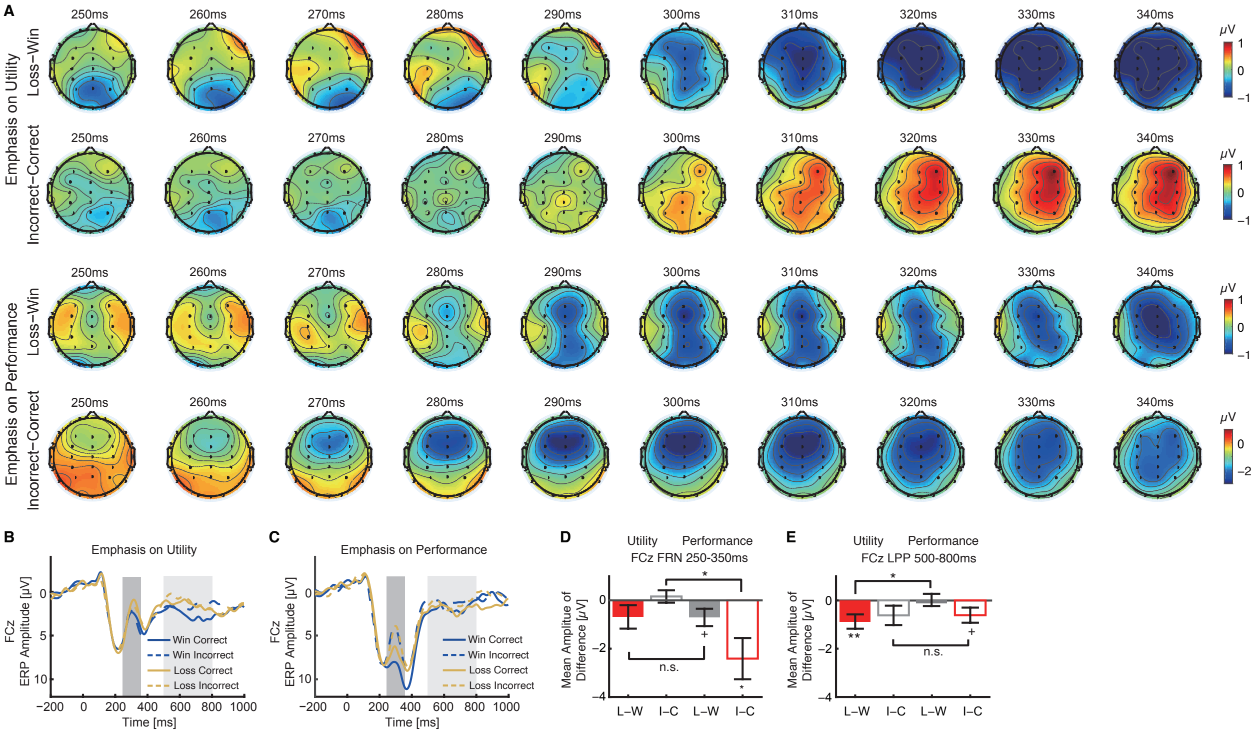

Supplementary Fig. 2. Control ERP results. **(A)** The scalp topography of the difference waveform between loss and win trials, and between incorrect and correct trials, in the FRN interval (250-350 ms). **(B-E)** ERP at electrode FCz. Gray shaded areas denote the FRN (250-350 ms) and LPP (500-800 ms) intervals. Blue: win. Yellow: loss. Solid line: correct. Dashed line: incorrect. **(B)** Emphasis on utility (win/loss). **(C)** Emphasis on performance (correct/incorrect). **(D)** Mean amplitude of difference for the FRN. **(E)** Mean amplitude of difference for the LPP. Error bars denote one SEM across participants. Asterisk indicates a significant difference using two-tailed one-sample t-test: +: P<0.1, *: P<0.05, and **: P<0.01. n.s.: not significant. Red: congruent / salient. Gray: incongruent / non-salient. Solid bars denote loss−win whereas open bars denote incorrect−correct.

Supplementary Fig. 3

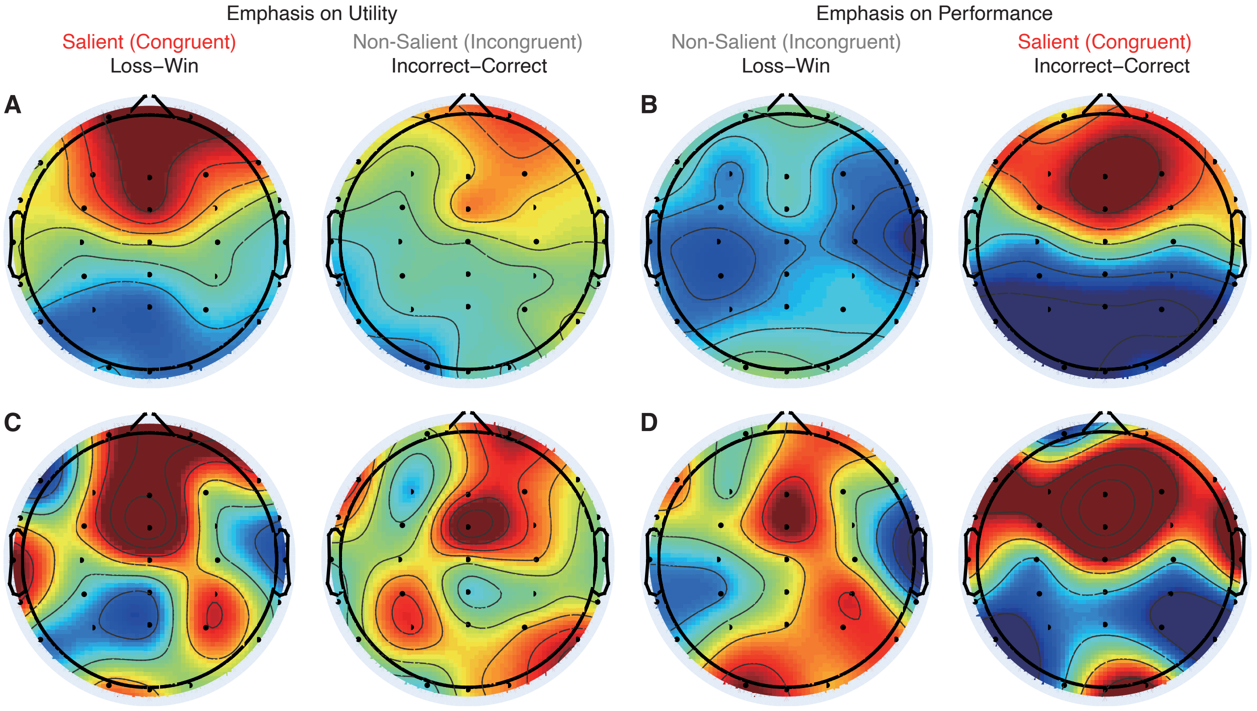

Supplementary Fig. 3. Topographic distribution of theta-band (4-7 Hz) power (250-500 ms). **(A, C)** Emphasis on utility. **(B, D)** Emphasis on performance. **(C, D)** Surface Laplacians[1] were computed to sharpen scalp topographies and eliminate volume-conducted contributions.

Supplementary References

1 Kayser, J. & Tenke, C. E. Principal components analysis of Laplacian waveforms as a generic method for identifying ERP generator patterns: I. Evaluation with auditory oddball tasks. *Clinical Neurophysiology* **117**, 348-368, doi:https://doi.org/10.1016/j.clinph.2005.08.034 (2006).
